# Comments on the Study of Aortomitral Continuity Calcification and Conduction Disturbances After TAVI

**DOI:** 10.1002/joa3.70195

**Published:** 2025-09-18

**Authors:** Hafsa Azam, Fatima Yaseen, Bariyah Ahmed, Maham Ejaz, Laiba Bibi

**Affiliations:** ^1^ Jinnah Sindh Medical University Karachi Pakistan

**Keywords:** aortomitral continuity calcification, conduction disturbances, transcatheter aortic valve implantation (TAVI)

## Abstract

Aortomitral continuity calcification (AMCC) may contribute to conduction disturbances after TAVI. This Letter highlights key methodological limitations including short‐term endpoints, omission of pre‐existing RBBB, and lack of spatial AMCC assessment that must be addressed to enhance the accuracy and clinical relevance of AMCC as a risk stratification tool.

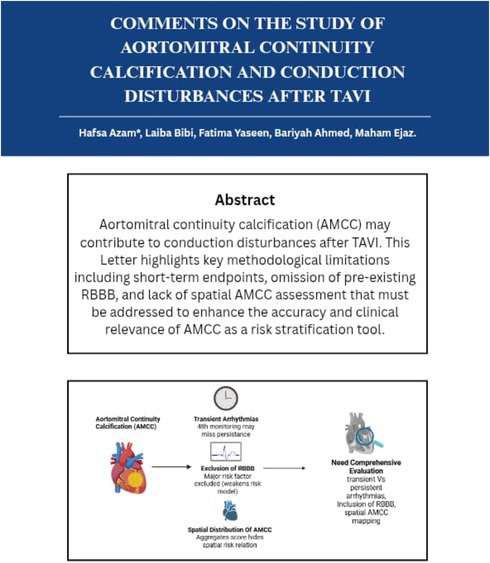

To Editor,

1

We read with interest the study by Aslan et al., which examined the relationship between aortomitral continuity calcification (AMCC) and conduction disturbances following transcatheter aortic valve implantation (TAVI) [1]. The authors present an important contribution by proposing AMCC as a potential imaging marker for conduction risk. Although the study contributes meaningful insights to an evolving field, several methodological and interpretive limitations should be addressed to improve the clinical relevance and generalizability of future investigations.

A key limitation of this investigation is its reliance on a binary composite endpoint of high‐grade atrioventricular block and permanent pacemaker insertion, without categorizing conduction disturbances by their timing. By focusing on a narrow 48‐hour period, the study cannot distinguish between transient and persistent conduction issues. When temporal resolution is limited, the clinical outcomes of these abnormalities become harder to derive meaningful conclusions, which complicates both interpretation and management. Newer evidence indicates that a considerable fraction of post‐TAVI conduction changes, notably newly appearing left bundle branch block and lengthened PR intervals, tend to be transient and often revert to baseline without intervention. In a prospective study by Sammour et al., approximately 45% of patients with new conduction disturbances recovered without requiring PPM, and persistent forms carried markedly different implications for pacing and prognosis [2]. Recognizing this dynamic behavior is essential to avoid confusing temporary adaptations with genuine conduction system injuries, which may clarify the relationship between aortomitral continuity calcification (AMCC) and significant clinical outcomes. Future analyses will benefit from incorporating continuous electrocardiographic monitoring to better categorize conduction disturbances, ultimately enhancing prognostic modeling and refining post‐TAVI pacing strategies.

The exclusion of pre‐existing right bundle branch blocks (RBBB) from the multivariable analysis due to low sample frequency compromises the clinical validity of the risk model. RBBB is not only a strong independent predictor of post‐TAVI conduction disturbances and permanent pacemaker (PPM) implantation but may also interact synergistically with anatomical substrates such as aortomitral continuity calcification (AMCC) [3]. Its omission limits the model's ability to account for baseline conduction vulnerability, thereby inflating the isolated effect size attributed to AMCC. This issue is not merely statistical but pathophysiological. The combination of pre‐existing RBBB and procedural stress on the left‐sided conduction system places the His‐Purkinje axis at high risk of complete block. Ignoring this high‐risk variable undermines both the accuracy and generalizability of the findings. Penalized regression approaches, such as Firth correction or LASSO, offer methodologically sound solutions for incorporating clinically important but infrequent covariates, and should be adopted in future TAVI risk models.

Although the authors mention that AMCC involved varying anatomical regions such as the right or left fibrous trigone or both, they do not analyze whether this spatial variability affects conduction outcomes [1]. All calcification is aggregated into a composite score, without considering its orientation or proximity to the conduction system. This limits the granularity of the analysis and may obscure critical anatomical patterns that contribute disproportionately to conduction disturbance risk. Calcification near the left fibrous trigone or membranous septum has been shown to exert focal mechanical stress on the His‐Purkinje system, increasing the likelihood of atrioventricular block independent of overall calcium burden [4]. Without accounting for topographic asymmetry or directional distribution, the study may understate the true mechanistic link between AMCC and post‐TAVI conduction abnormalities. Future models should incorporate spatially resolved imaging markers such as proximity‐weighted scoring or directional segmentation to improve the specificity of AMCC‐based risk stratification.

All these points highlight the need for more thorough anatomical analyses and better methods, which will lead to more insightful studies and enhance how effectively AMCC can predict conduction risk after TAVI.

## Ethics Statement

The authors have nothing to report.

## Consent

The authors have nothing to report.

## Conflicts of Interest

The authors declare no conflicts of interest.

## Data Availability

The authors declare no conflicts of interest.
